# Prevalence of Intestinal Parasitic Infections and Associated Risk Factors among the First-Cycle Primary Schoolchildren in Sasiga District, Southwest Ethiopia

**DOI:** 10.1155/2020/8681247

**Published:** 2020-03-13

**Authors:** Baye Sitotaw, Wakgari Shiferaw

**Affiliations:** ^1^Department of Biology, Bahir Dar University, Ethiopia; ^2^ONRS, Sasiga District Education Office, Ethiopia

## Abstract

Intestinal parasitic infections (IPIs) have been major public health burdens in low-income countries like Ethiopia. Studies in different areas of Ethiopia have shown a high prevalence of IPIs in poor families. A similar study has not been conducted in Sasiga District given that the area is possibly at high-risk of IPIs due to the prevailing risk factors. This study is aimed at assessing the prevalence of IPIs and associated risk factors among schoolchildren in Sasiga District, southwest Ethiopia. A school-based cross-sectional study was conducted from December 2018 to March 2019 to estimate the prevalence of IPIs and associated risk factors among the study participants. A total of 383 children were selected using resident-type and grade-level stratified systematic random sampling technique. Stool samples were examined microscopically using direct wet mount and formal-ether concentration techniques. A structured questionnaire was used to get information on the associated risk factors. Data were analyzed using SPSS version 20 and *p* value of ≤0.05 was taken as statistically significant. The overall prevalence of IPIs among the children was 62.4% (239/383). Single, double, and triple infections were 49.9%, 10.7%, and 1.83%, respectively. Residence, family income, place of defecation, source of drinking water, shoe-wearing habit, handwashing habit after toilet use, ways of waste disposal, and cleanliness of fingernail were the most important predictors of IPIs (*p* < 0.05). *Ascaris lumbricoides* (22.7% (87/383)) and hookworms (20.6% (79/383)) were the most prevalent parasites, followed by *Entamoeba histolytica* (8.1%), *Trichuris trichiura* (7.6%), *Giardia intestinalis* (6.5%), *Hymenolepis nana* (5.7%), and *Schistosoma mansoni* (4.4%), in that order. Sasiga District primary schoolchildren are likely at a high burden of IPIs. Intensive health education on personal hygiene and environmental sanitation is needed.

## 1. Introduction

Intestinal parasitic infections (IPIs) have been a big concern for low-income countries as they are the major cause of high morbidity and mortality. Most infectious diseases caused by members of the intestinal parasites (protozoan and helminths) have been considered as Neglected Tropical Diseases (NTDs) [1–4] and are affecting a large section of poor communities. Intestinal helminthiasis and protozoan infections are widespread throughout the world [5–7], and in particular, millions of people in low-income countries are infected and/or ill with parasitic infections (PIs). Due to this, ending epidemics of NTDS through the control of the transmission of IPIs and the mitigation of possible risk factors is one of the sustainable development goals (SDG) of the United Nations (2030 Agenda; Goal 3.3).

Regardless of huge budget mobilization on health improvement following SDG and significant improvements on the diagnosis of parasitic diseases and subsequent treatments [8, 9], any reduction has not shown, and IPIs happen to be the major public health problem in low-income countries primarily affecting schoolchildren [10–25]. Factors related to poverty, lack of awareness, and unavailability of sufficient health care as well as the prevailing bad climatic and environmental conditions are the most aggravating risk factors for IPIs.

Children are affected by IPIs far more than adults due to their higher nutritional requirements and less developed immune systems. In children, IPIs affect growth rate, protein-energy balance, and iron availability and consequently reduce mental development [1, 26, 27]. Globally, millions of preschoolers and schoolchildren are vulnerable to infections by parasitic worms and pathogenic protozoan species [1, 3, 4] and are demanding urgent treatment and preventive interventions.

The protozoan parasite (*Entamoeba histolytica* and *Giardia intestinalis/lamblia*) and soil-transmitted helminths (*Ascaris lumbricoides*, *Trichuris trichiura*, and hookworm) are the most prevalent intestinal parasites causing high morbidity and mortality in sub-Saharan Africa, affecting nearly all inhabitants at some point in their lives [1, 10, 13, 15, 18–25]. The prevalence of IPIs in the region is reported to be as high as 84% in Ethiopia [20], 90% in Central Sudan [28], and 84.7% in Burkina Faso [29].

To accelerate the country's progress in meeting some of the Millennium Development Goals, the Federal Ministry of Health of Ethiopia started the health extension program since 2004. The health extension workers have been trained and assigned at almost every village in rural as well as urban areas. Despite the efforts, Ethiopia is still at a high burden of IPIs due to the aforementioned sociodemographic variables, behavioral factors, personal hygiene, and environmental sanitation factors [1, 18–26, 29–32]. Particularly, ascariasis, hookworm, and trichuriasis are listed among the most common public health burdens in Ethiopia [3]. Studies in the different regions of Ethiopia have shown a considerably high prevalence of IPIs. For instance, an extremely high prevalence (84%) was reported among Debre Elias primary school children (northwest Ethiopia) [20]. In other studies conducted in the different regions of Ethiopia, the overall prevalence of IPIs, ranging from 54.5% to 83%, was reported from primary schoolchildren [18, 19, 21–25, 30–32]. To get a deeper insight into the magnitude of the problem and design effective intervention mechanisms, more information is needed from different localities where similar studies have not been conducted.

Clinical and health center reports (from years 2015 to 2017) in Sasiga District indicated that IPI was the main reason why many people visit health facilities. However, there was no scientific study conducted on the prevalence of IPIs and associated risk factors among schoolchildren in the study area. Therefore, the objective of this study was to estimate the prevalence of IPIs and associated risk factors among the first-cycle primary schoolchildren in Sasiga District, southwest Ethiopia.

## 2. Methods

### 2.1. Study Design and Study Area

A school-based cross-sectional study was conducted from December 2018 to March 2019 to determine the prevalence of IPIs and the associated risk factors among the first-cycle primary schoolchildren in Sasiga District, southwest Ethiopia. Sasiga District is located 346 km southwest of Addis Ababa (the capital city of Ethiopia). The district is located at the geographical location of 9°10′N and 36°30′E and elevations of about 1,742 to 2,034 meters above sea level. Sixty percent of the district is midland to highland while 40% is a lowland agroecological zone. The annual mean temperature in the district ranges from 25°C to 32°C while the annual rainfall ranges from 1,200 mm to 1,800 mm.

Based on the 2015 national housing and population census, the total population of the district was 105,603 (53,990 males and 51,613 females) (Sasiga District Administration Office, 2018). In Sasiga District, there are 6 governmental health centers, 14 private clinics, and 32 community health posts with a total of 187 health workers. In this district, access to clean water was low; as a result, people were forced to use various unprotected water sources such as rivers (authors' observation).

### 2.2. Study Population and Sample Size Determination

Based on the information from Sasiga District Educational Office (2018), there were 12 first-cycle (grade 1 to 4) primary schools, 30 first and second-cycle (grade 1 to 8) primary schools, and 7 secondary schools in the district. The study population was all schoolchildren enrolled in the first-cycle primary schools in Sasiga District (from grades 1 to 4). And the study participants were all students who are volunteers (gave consent) to participate in the study and those who did not take any antiintestinal parasitic drugs within 2 weeks. Of the 12 first-cycle primary schools in the district, 4 schools (2 from urban and 2 from rural) were selected for this study. There were 878 students enrolled in these 4 schools in the 2018/2019 academic year. Since there was no similar study previously conducted in the area, a 50% prevalence rate of IPIs was taken assuming that IPIs are significantly prevalent among students in the first-cycle primary schools in Sasiga District. Accordingly, the minimum number of sample size (*n*) required was determined using a single population proportion formula for cross-sectional surveys [21], i.e., *n* = *Z*^2^ p (1 − p)/*d*^2^ = 1.96^2^ × 0.50 × 0.50/.05^2^ = 384 students. To compensate for the nonrespondents and to minimize errors probably arising from the likelihood of noncompliance, 10% was added giving a final sample size of 422 study participants.

Study participants were selected from the students stratified into four schools using a quota system. Again, quota system was used to select students from the four grade levels at each school. The actual number of students participated in the study from each grade level was selected by systematic random sampling technique to include 422 schoolchildren.

### 2.3. Sample Collection and Processing

A structured questionnaire based on known risk factors was developed in English and translated into Oromo language (local language). The participants of the study or parents in the case of younger children were interviewed to obtain information on sociodemographic characteristics, behavioral factors, hygienic practices, and environmental sanitation factors (see Table 1 and additional [Supplementary-material supplementary-material-1] for the details). Then, the responses were translated back into English. The questionnaire was pretested using forty individuals outside the study area in a nonstudy sample population.

For parasitological analysis, fresh stool samples were collected from each schoolchild. The children were instructed properly and were given clean labeled collection cups along with applicator sticks, and from each student, about 2 g of fresh stool was collected. At the time of collection, date of sampling, school name, the name of the participant, age, and sex were recorded for each subject in a recording format. Stool samples were preserved in a 10% formalin before transported to the health center laboratory. A portion of each of the stool samples was processed and examined microscopically using direct wet mount and formal-ether concentration techniques following the procedures in WHO guidelines [33]. All developmental stages of the parasites (cyst, egg, larvae, and adult) were recorded.

### 2.4. Limitations of the Study

The study was limited to only students in the first-cycle of primary school. Including second-cycle primary schools would have been better to get a bigger picture of the prevalence of IPIs among schoolchildren of the area. The study was also limited to the presence or absence of infections without quantifying the parasite load, which may not show that the infected students were diseased. Moreover, a self-reported data collection method was used which may also bias the information.

### 2.5. Data Analysis

Statistical Package for Social Science (SPSS) software version 20 was used to analyze the collected data. Chi-square (*χ*^2^) test was performed to verify the possible association between the prevalence of IPIs and sociodemographic characteristics, behavioral factors, hygienic practices, and environmental sanitation factors. Logistic regression was used to measure the strengths of association between the prevalence of infection and the risk factors using odds ratio. In the modeling process, a univariate analysis (crude odds ration) was first done with a 0.25 level of significance to select the candidate variables for multivariate analysis (adjusted odds ratio). The variables, significant at the univariate analysis, were then included in the multivariate analysis [34]. Values were considered significant at *p* ≤ 0.05.

## 3. Results

### 3.1. Sociodemographic Characteristics of the Study Participants

From a total of 422 students selected for these study, 39 (10.2%) were excluded due to either incomplete information or insufficient fecal specimen production. As a result, 383 (90.76%) gave stool for intestinal parasitic examination and filled questionnaires on associated risk factors. From 383 study participants, about half were urban dwellers; female participants were slightly greater (51.2%) than males (48.8%); most were from farmer father (87%) and housewife mother (91%), and most were from families who earned Birr 800-2,000 monthly (Table 1). The age of the participants ranged from 5 to 15 years, and most of the participants (83.3%) were 6-11 years old. Similarly, most of the participants were from mothers who attended only primary school (90.6%) and a little more than half (57.7%) from fathers who attended only primary school. Furthermore, most of the participants (91%) came from family sizes of 5 and above (Table 1).

### 3.2. Prevalence of Intestinal Parasitic Infections among the Study Participants

Three hundred eighty-three students were examined for IPIs, and 62.40% of these participants were positive for at least one intestinal parasite. The rates of single, double, and triple infections were 191 (49.9%), 41 (10.7%), and 7 (1.83%), respectively. The prevalences of protozoa, helminths, and mixed infections were 26 (6.8%), 181 (47.26%), and 32 (8.36%), respectively. Identified intestinal parasites, in order of decreasing prevalence rate, were *Ascaris lumbricoides* (22.7% (87/383)) and Hookworms (20.6% (79/383)), *Entamoeba histolytica* (8.1%), *Trichuris trichiura* (7.6%), *Giardia intestinalis* (6.5%), *Hymenolepis nana* (5.7%), and *Schistosoma mansoni* (4.4%) (Table 2).

### 3.3. Association of the Different Risk Factors with Intestinal Parasitic Infections

The overall infection rate was similar in males (62.6%) and females (62.2%). Moreover, there was no significant variation in the infection rate of IPIs among the students in the different age groups, grade level, and family size (*p* > 0.05). However, there was a statistically significant association between IPIs and some other sociodemographic factors including residence, family income, educational status, and occupation of the parents. Accordingly, a significantly higher prevalence rate of IPIs was observed among students living in rural (76.4%) than urban (49.8%) areas. Similarly, students from families who earned less than Birr 800 (Ethiopian currency) were more infected with IPs (86.5%) compared with families who earned more than Birr 800 (44%) (*p* < 0.05). Likewise, students from families who attended only primary school were more infected (64.5-71.1%) with IPs compared with those from families who attended secondary school and above (41.7-50.6%). Types of mother and father occupation had also a significant effect on the infection rate of IPIs (Supplementary [Supplementary-material supplementary-material-1]).

Except for the habits of eating raw meat, all other factors related to the practices in personal hygiene and environmental sanitation had significant effects on the rate of IPIs among the students (Supplementary [Supplementary-material supplementary-material-1]). The prevalence of IPIs among the participants who had varying habits (did always, sometimes, or not all) of shoe wearing, hand washing after toilet use, and eating unwashed fruits and vegetables was statistically significant (*p* < 0.002). Similarly, cleanliness of students' fingernails (clean or unclean), place of defecation (open-field or in latrine), source of drinking water (river, well, or pipe), and ways of waste disposal (open dump, burry, or burn) had significant effects on the prevalence rate of IPIs among the participants (*p* < 0.002). Consequently, high prevalence rates of IPIs were observed in children who did not wear shoe at all and wore sometimes, who did not wash their hands always after toilet use, who had habits of open-field defecation, who ate unwashed fruits and vegetables, and who had dirty fingernails (close observation by the investigator) (Supplementary [Supplementary-material supplementary-material-1]).

### 3.4. Logistic Regression Analysis (LRA) of the Most Important Risk Factors for IPIs

The most important risk factors for IPIs among the first-cycle primary schoolchildren in Sasiga District were identified using Multivariable Logistic Regression Analyses (MLRA) (Table 3). In the modeling process, a univariate analysis was first done with a 0.25 level of significance to select the candidate variables for multivariable analysis. Sixteen (out of eighteen) variables, significant at the univariate analysis, were included in the multivariable analysis [34]. Residence, family income, father occupation, handwashing habit after toilet use, place of defecation, source of drinking water, habit of wearing shoes, ways of waste disposal, and unclean fingernails were significantly associated (*p* < 0.05) with IPIs (Table 3) and were found to be the most important predictors of IPIs among the students in the first-cycle primary school in Sasiga District.

Accordingly, the likelihood of being infected by intestinal parasites (IPs) was increased by fivefold (AOR = 5.45, CI = 2.293 − 12.949; *p* ≤ 0.001) in students who lived in rural area than in urban area (Table 3); the risk of being infected by IPs was increased by 4 times (AOR = 4.159; CI = 1.257 − 13.764; *p* = 0.02) in students whose parents' monthly income was less than Birr 800 compared to students whose parents' income was more than Birr 2,000; the likelihood of being infected by IPs was increased eightfold (AOR = 8.377; CI = 3.552 − 19.752; *p* ≤ 0.001) and 12-fold (AOR = 12.127; CI = 4.247 − 34.626; *p* ≤ 0.001) in students who buried wastes and dumped wastes in open field, respectively, than students who burned the wastes; the risk of being infected by IPs was increased by almost fivefold in students who defecated in open field (AOR = 4.747; CI = 1.820 − 12.379; *p* = 0.001) than those who used latrine; students who did not wear shoes were about 7 times (AOR = 6.889; CI = 3.518 − 13.489; *p* ≤ 0.001) more likely to be infected with IPs than those who always wore shoes; students who did not regularly wash their hands after toilet use were about 2 times (AOR = 2.358; CI = 1.047 − 5.312; *p* = 0.038) more likely to have IPs than those who washed their hands regularly after toilet use; students who used river as source of drinking water were 3 times (AOR = 3.124; CI = 1.325 − 7.362; *p* = 0.009) more likely to have IPIs than those who used tap water for the same purpose; students who had unclean fingernails were more likely to have IPIs than those students who had clean fingernails (Table 3).

### 3.5. Risk Factors Associated with the Prevalence of Individual Parasites

Five helminths and two protozoan intestinal parasitic species were identified in this study, of which infections *by Ascaris lumbricoides and* Hookworm were the most prevalent (Table 2). Infections by the different parasites were associated with some particular groups of risk factors for each parasite (Table 2 and Supplementary [Supplementary-material supplementary-material-1]). In the MLRA model, residence, family size, and ways of waste disposal were predictors of *A. lumbricoides* infection. Accordingly, the risk of *A. lumbricoides* infection was increased by about threefold in students from the family size of less than 4 (AOR = 2.706; CI = 1.043 − 7.017; *p* = 0.041) compared with those from the family size of 8 and above (Table 4). Similarly, the likelihood of being infected by *A. lumbricoides* was increased eightfold (AOR = 8.033; CI = 3.022 − 22.134; *p* ≤ 0.001) and fivefold (AOR = 4.898; CI = 1.722 − 13.929; *p* = 0.03) in students who dumped wastes in open-field and buried wastes, respectively, than students who burned the wastes. Shoe-wearing habits and ways of waste disposal were predictors of hookworm infection among the study participants. Students who did not wear shoes were 8 times (AOR = 8.346; CI = 2.908 − 18.761; *p* = 0.001) and who sometimes wore shoes were 3 times (AOR = 3.648; CI = 2.094 − 6.358; *p* = 0.001) more likely to be infected with hookworm compared with students who always wore shoes. And students who buried wastes were about 3 times (AOR = 2.97; CI = 1.29 − 6.838; *p* = 0.011) more likely to be infected with hookworm compared to those who burned the wastes. As indicated in the Supplementary [Supplementary-material supplementary-material-1], residence, raw meat-eating habits, and age were found to be predictors of infections by *Trichuris trichiura*, *Hymenolepis nana*, and *Schistosoma mansoni*, respectively.

## 4. Discussion

IPIs continue to challenge public health in low-income countries like Ethiopia. To identify high-risk communities and design effective intervention mechanisms, studies in different settings are a vital step. Based on this view, we estimated the prevalence of IPIs and identified associated risk factors among students attending the first-cycle primary schools in Sasiga District, southwest Ethiopia.

The overall prevalence of IPIs among the study participants was notably high (62.4%), showing that this community is likely at a high burden of intestinal parasitosis given methodological limitations. Similarly, high-risk communities, especially school-aged children, have been shown through cross-sectional studies conducted in the different parts of Ethiopia [18, 20–25, 31, 32, 35–37]. Even, extreme prevalence rates (over 75%) were reported from different regions of Ethiopia [18, 20, 21, 32]. The community-based accelerated expansion of health facilities in Ethiopia being operational since 2004 seems ineffective as a sound reduction in the prevalence of such neglected diseases was expected. Parts of communities in other low-income countries such as India (43-49%) [12, 38], Nepal (52-59%) [39, 40], Nigeria (58.3-81%) [15, 41, 42], Burkina Faso (65-84.7%) [13, 43], and Peru (100%) [16] are also reported to be at high-risk of IPIs indicating that IPIs continue to be major threats to poor society. Low socioeconomic status, low educational level, and hence poor knowledge, attitude, and practices towards easily preventable disease, poor personal hygiene, and environmental sanitation, lack of potable, and sufficient drinking water are the most important risk factors for the high infection rate of intestinal parasites among poor communities.

In this study, the infection rate was strongly associated (*p* ≤ 0.009) with 13 out of 18 risk factors considered (Supplementary [Supplementary-material supplementary-material-1]). All these factors are already documented elsewhere as the main predictors of IPIs. Low level of knowledge and practices in personal hygiene and environmental sanitation due to lack of access to education, low living standards of the community, and inadequate and unsafe water supply is often recognized as a major factor for the high prevalence of intestinal parasites among such communities.

On the contrary, sex, age, family size, grade level, and raw meat-eating habits were not associated with IPIs. In many studies, males were found to be more exposed to IPIs than females [23, 24, 37, 44] due to differences in gender roles. In this study, however, male and female participants were found to be equally infected with intestinal parasites (Additional [Supplementary-material supplementary-material-1]). Previous studies in Jawi [25] and Tilili [36] towns (northwest Ethiopia) and Babile town [45] (Southern Ethiopia) have also shown similar observations. This may indicate that the differences in gender roles are narrowing. Regarding age, most of the participants (83.5%) were within a similar age group (midchildhood), and as a result, the differences may not be expected within this narrow age range.

Regarding individual parasites identified, almost half of the participants were infected with parasitic helminths predominantly by *Ascaris lumbricoides* (close to 23%) and Hookworms (about 21%). *A. lumbricoides* infection is a worldwide problem mainly in tropical and subtropical countries where there are conducive conditions [46, 47]. Such a high prevalence rate of *A. lumbricoides* among students in the first-cycle primary schools in Sasiga District may be attributed to one or more of the risk factors. The area is characterized mostly by moist, warm, and shaded soils which are suitable conditions for the parasite. Moreover, a significant proportion of the participants were from the rural area (47%) and who dumped wastes in open area (39%) both of which can be contributing factors as also shown in MVLRA (Table 4). *A. lumbricoides* was also found to be highly prevalent (11-23%) among other schoolchildren in Ethiopia [18, 24, 32]. Hookworm infection has also been a global health problem and more prevalent in low-income countries [48–50]. Hookworm infection is also reported to be high (11-33%) among schoolchildren in the different regions of Ethiopia [18, 21, 22, 25, 32, 37]. Walking barefoot in a warm climate, poor personal hygiene, and environmental sanitation are very important risk factors for hookworm infection. In this study, hookworm infection was significantly associated with shoe-wearing habits, ways of waste disposal, defecation habit, source of drinking water, fingernail cleanliness, and handwashing habit after toilet use (Tables 2 and 4 and Supplementary [Supplementary-material supplementary-material-1]).

Infection rates by the rest five parasites (identified in this study) were relatively low as compared to the reports from different parts of Ethiopia. However, *S. mansoni* infection cannot be underestimated due to its seriousness. About 17 (4.4%) schoolchildren were found to be infected with *S. mansoni*, and the most important risk factor was age. Older children were more likely to get *S. mansoni* infection than younger ones (Supplementary [Supplementary-material supplementary-material-1]). In such communities, older children are more commonly engaged in field activities compared to youngsters that may expose them to *S. mansoni* infection.

## 5. Conclusion

Based on the result of this study, children attending in the first-cycle primary schools in Sasiga District are likely at high-risk of IPIs, showing that the burden of IPIs continues to be endemic in poor communities. *A. lumbricoides* and hookworm were the most prevalent intestinal parasites among the schoolchildren in the study area. Living in rural area, low family income, poor handwashing habit after toilet use, open-field defecation habit, lack of access to safe drinking water, inconsistence of shoe-wearing habit, inappropriate waste disposal methods, and unclean fingernails were found to be the most important predictors of IPIs among the students in the first-cycle primary school in Sasiga District. To reduce such a high burden of intestinal parasitic infections, effective strategies should be designed and implemented that involve decision makers, health workers, school teachers, the mass media, and community and religious leaders.

## Figures and Tables

**Table 1:** Sociodemographic characteristics of the study participants.

Sociodemographic variables	Categories	Frequency	Percentage
Grade level	1	120	31.3
	2	112	29.2
	3	83	21.7
	4	68	17.8

Age groups (years)	Childhood (<5)	8	2.1
	Mid childhood (6-11)	320	83.5
	Early adolescent (12-18)	55	14.4

Sex	Male	187	48.8
	Female	196	51.2

Residence	Urban	201	52.5
	Rural	182	47.5

Family size	<4	45	11.75
	5-7	191	49.9
	8-10	147	38.4

Family monthly income (ETB)^∗^	<800	163	42.6
	800-2,000	170	44.9
	>2,000	50	13.1

Father education	Primary school	221	57.7
	Secondary school and above	162	42.3

Mother education	Primary school	347	90.6
	Secondary school and above	36	9.44

Father occupation	Farmer	332	86.7
	Government employee	16	4.2
	Merchant	20	5.2
	Daily laborer	15	3.9

Mother occupation	Housewife	350	91.4
	Government employee	9	1.3
	Merchant	21	5.5
	Daily laborer	3	1.6

^∗^1 USD is about 30 ETB.

**Table 2:** Association of individual intestinal parasites with sociodemographic variables.

Intestinal parasites	1	2	3	4	Total (%)	*χ* ^2^	*p* value
	Grade level no. (%)						
*A. lumbricoides*	32 (26.7)	21 (18.8)	18 (21.7)	16 (23.5)	87 (22.7)	2.146	0.543
Hookworms	28 (23.3)	25 (22.3)	11 (13.3)	15 (22.1)	79 (20.6)	3.575^a^	0.311
*E. histolytica*	11 (9.2)	10 (8.9)	5 (6)	5 (7.4)	31 (8.1)	0.819	0.845
*T. trichiura*	10 (8.3)	6 (5.4)	8 (9.6)	5 (7.4)	29 (7.6)	1.396	0.707
*G. intestinalis*	10 (8.3)	12 (10.7)	1 (1.2)	2 (2.9)	25 (6.5)	9.147	0.027^∗^
*H. nana*	5 (4.2)	10 (8.9)	4 (4.8)	3 (4.4)	22 (5.7)	3.003	0.391
*S. mansoni*	2 (1.7)	6 (5.4)	4 (4.8)	5 (7.4)	17 (4.4)	3.787	0.285
	Age in years no. (%)						
	≤5 (%)	6-11 (%).	12-18 (%)				
*A. lumbricoides*	4 (50)	65 (20.3)	18 (32.7)	-	87 (22.7)	7.585	0.023^∗^
Hookworms	2 (25)	67 (20.9)	10 (18.2)	-	79 (20.6)	0.313	0.855
*E. histolytica*	1 (12.5)	29 (9.1)	1 (1.8)	-	31 (8.1)	3.524	0.172
*T. trichiura*	-	25 (6.5)	4 (7.4)	-	29 (7.6)	0.689	0.709
*G. intestinalis*	1 (12.5)	22 (6.9)	2 (3.6)	-	25 (6.5)	1.285	0.526
*H. nana*	-	20 (6.2)	2 (3.6)	-	22 (5.7)	1.090)	0.58
*S. mansoni*	-	11 (3.4)	6 (10.9)	-	17 (4.4)	6.556	0.038^∗^
	Sex no. (%)						
	Male	Female					
*A. lumbricoides*	41 (21.9)	46 (23.5)	-	-	87 (22.7)	0.13	0.718
Hookworms	37 (19.8)	42 (21.4)	-	-	79 (20.6)	0.158	0.691
*E. histolytica*	13 (7.0)	18 (9.2)	-	-	31 (8.1)	0.641	0.423
*T. trichiura*	17 (9.1)	12 (6.1)	-	-	29 (6.7)	1.205)	0.272
*G. intestinalis*	11 (8.9)	14 (7.1)	-	-	25 (6.5)	0.249	0.618
*H. nana*	14 (7.5)	8 (4.1)	-	-	22 (5.7)	2.049	0.152
*S. mansoni*	7 (3.7)	10 (5.1)	-	-	17 (4.4)	0.417	0.519
	Residence no. (%)						
	Urban (%)	Rural (%)					
*A. lumbricoides*	41 (20.4)	46 (25.3)			87 (22.7)	1.294	0.255
Hookworms	41 (20.4)	38 (20.9)			79 (20.6)	0.014	0.907
*E. histolytica*	20 (10)	11 (6)			31 (8.1)	1.959	0.162
*T. trichiura*	6 (3)	23 (12.6)			29 (7.6)	12.72	0.001^∗∗^
*G. intestinalis*	10 (5)	15 (8.2)			25 (6.5)	1.67	0.196
*H. nana*	0	22 (12.1)			22 (5.7)	25.78	0.001^∗∗^
*S. mansoni*	10 (5)	7 (3.8)			17 (4.4)	0.278	0.592
	Family size no. (%)						
	2-4	5-7	8-10				
*A. lumbricoides*	12 (26.7)	51 (26.7)	24 (16.3)	-	87 (22.7)	5.547	0.062
Hookworms	5 (11.1)	43 (22.5)	31 (21.1)	-	79 (20.6)	2.923	0.232
*E. histolytica*	1	24 (12.6)	6 (4.1)	-	31 (8.1)	2.106	0.147
*T. trichiura*	4 (8.9)	12 (6.3)	13 (8.8)	-	29 (7.6)	0.905	0.636
*G. intestinalis*	1	12 (6.3)	12 (8.2)	-	25 (6.5)	2.030	0.362
*H. nana*	3 (6.7)	13 (6.8)	6 (4.1)	-	22 (5.7)	1.219	0.544
*S. mansoni*	2 (4.4)	7 (3.7)	8 (5.4)		17 (4.4)	0.346	0.556
	Family monthly income (ETB) no. (%)						
	≤800 (%)	800-2,000 (%)	≥2,000 (%)				
*A. lumbricoides*	57 (35)	23 (13.5)	7 (14)		87 (22.7)	24.28	0.001^∗∗^
Hookworms	41 (25)	31 (18)	7 (14)		79 (20.6)	3.975	0.137
*E. histolytica*	21 (12.9)	8 (4.7)	2 (4)		31 (8.1)	8.776	0.012^∗^
*T. trichiura*	19 (11.7)	9 (5.3)	1 (2)		29 (7.6)	7.364	0.025^∗^
*G. intestinalis*	13 (8)	8 (4.7)	4 (8)		25 (6.5)	1.662	0.436
*H. nana*	14 (8)	6 (3.5)	2 (4)		22 (5.7)	4.258	0.119
*S. mansoni*	9 (2.3)	5 (1.3)	3 (0.8)		17 (4.4)	1.637	0.441
	Father education no. (%)						
	Primary school	Secondary school and above					
*A. lumbricoides*	60 (27.1)	27 (17)	-		87 (22.7)	7.009	0.03^∗^
Hookworms	54 (24.4)	25 (16)	-		79 (20.6)	5.399	0.067
*E. histolytica*	25 (11.3)	6 (3.9)	-		31 (8.1)	7.409	0.025^∗^
*T. trichiura*	17 (7.7)	12 (7.4)	-		29 (7.6)	0.505	0.777
*G. intestinalis*	12 (5.4)	13 (8)	-		25 (6.1)	1.502	0.472
*H. nana*	12 (5.4)	10 (6.5)	-		22 (5.7)	0.61	0.737
*S. mansoni*	13 (5.9)	4 (2.6)	-		17 (4.4)	2.673	0.263
	Mother education no. (%)						
	Primary school	Secondary school and above					
*A. lumbricoides*	80 (23.1)	7 (21.9)	-		87 (22.7)	1.211	0.546
Hookworms	76 (19.8)	3 (9.4)	-		79 (20.6)	3.859	0.145
*E. histolytica*	30 (8.6)	1 (3.1)	-		31 (8.1)	1.556)	0.459
*T. trichiura*	24 (6.9)	1 (3.1)	-		25 (6.5)	0.973	0.615
*G. intestinalis*	21 (6.1)	1 (3.1)	-		22 (5.7)	0.71	0.701
*H. nana*	17 (4.9)	0 (0.00)	-		17 (4.4)	1.211	0.807
*S. mansoni*	27 (7)	2 (0.5)	-		29 (7.6)	0.429	0.807
	Father occupation no. (%)						
	Daily laborer	Farmer	Govt. employee	Merchant			
*A. lumbricoides*	9 (60)	73 (22)	1 (6.2)	4 (20)	87 (22.7)	14.53	0.002^∗^
Hookworms	5 (33.3)	67 (20.2)	3 (18.8)	4 (20)	79 (20.6)	1.559	0.669
*E. histolytica*	3 (20)	27 (8.1)	-	1 (6.7)	31	4.525	0.21
*T. trichiura*	1 (6.7)	21 (6.3)	1 (6.2)	2 (10)	25 (6.5)	0.42	0.936
*G. intestinalis*	1 (6.7)	20 (6)	-	1 (5)	22 (5.7)	1.067	0.785
*H. nana*	-	16 (4.8)	-	1 (5)	17 (4.4)	1.586	0.667
*S. mansoni*	5 (33.5)	23 (6.9)	1 (6.2)	-	29 (7.6)	16.1	0.001^∗∗^
	Mother occupation no. (%)						
	Daily laborer	Housewife	Govt. employee	Merchant			
*A. lumbricoides*	1 (33.3)	82 (23.4)	-	4 (19)	87 (22.7)	3.1	0.376
Hookworms	1 (33.3)	76 (21.7)	-	2 (9.5)	79 (20.6)	4.469	0.215
*E. histolytica*	2 (66.7)	27 (7.7)	-	2 (0.5)	31 (8.1)	14.75	0.002^∗^
*T. trichiura*	-	25 (7.1)	-	-	25 (6.5)	2.522	0.471
*G. intestinalis*	1 (33.3)	20 (5.7)	-	1 (4.8)	22 (5.7).	4.804	0.187
*H. nana*	-	17 (4.9)	-	-	17 (4.4)	1.677	0.642
*S. mansoni*	-	28 (8)	-	1 (4.8)	29 (7.6)	1.312	0.721
Single infection	-	-	-	-	191 (49.9)	-	-
Double infection	-	-	-	-	41 (10.7)	-	-
Triple infection	-	-	-	-	7 (1.8)	-	-
Overall infection	-	-	-	-	239 (62.4)		

^∗∗^Statistically significant at *p* ≤ 0.001. ^∗^Statistically significant at *p* < 0.05.

**Table 3:** Risk factors associated with overall intestinal parasitic infections.

Sociodemographic variables						
Number and percentage of parasite-infected student						
Variables	Categories	Total no. (%)	Positive no. (%)	Negative no. (%)	AOR (95% CI)	*p* value
Grade level	1	120 (31.3)	80 (66.6)	40 (33.33)	0.878 (0.28-2.6)	0.817
	2	112 (29.2)	71 (63.4)	41 (36.6)	1.379 (0.45-4.2)	0.571
	3	83 (21.7)	44 (53)	39 (47)	0.961 (0.28-3.2)	0.949
	4	68 (17.8)	44 (64.7)	24 (35.3)	1	
Residence	Rural	182 (47.5)	139 (76.4)	43 (23.6)	5.41 (2.3-12.9)	0.000^∗∗^
	Urban	201 (52.5)	100 (49.8)	101 (50.2)	1	
Family size	2-4	45 (11.75)	23 (51.1)	22 (48.9)	1.865 (0.5-6.31)	0.316
	5-7	191 (49.9)	129 (67.5)	62 (32.5)	1.389 (0.61-3.1)	0.429
	8-10	147 (38.38)	87 (59.2)	60 (40.8)	1	
Family monthly income (ETB)	<800	163 (42.6)	141 (86.5)	22 (13.5)	4.159 (1.25-13.76)	0.02^∗^
	800-2,000	170 (44.9)	76 (44.7)	94 (55.3)	1.383 (0.4-4.17)	0.585
	>2.000	50 (13.1)	22 (44)	28 (56)	1	
Father education	Primary school	221 (57.7)	157 (71.1)	64 (28.9)	1.229 (0.5-2.64)	0.598
	Secondary school and above	162 (42.3)	82 (50.6)	80 (49.4)	1	
Mother education	Primary school	347 (90.6)	224 (64.5)	123 (35.5)	0.990 (0.22-4.35)	0.990
	Secondary school and above	36 (9.44)	15 (41.7)	21 (58.3)	1	
Fathers' occupation	Farmer	332 (86.7)	208 (62.6)	124 (37.4)	0.624 (0.19-2.05)	0.438
	Others	51 (13.3)	31 (60.78)	20 (21.21)	1	
Mothers' occupation	Housewife	350 (91.4)	229 (65.4)	121 (34.6)	1.88 (0.368-9.67)	0.447
	Others	33 (8.6)	10 (30.3)	23 (67.7)	1	

Behavioral factors, hygienic practice, and environmental variables						
Place of defecation	Open field	148 (38.6)	139 (93.9)	9 (6.1)	4.747 (1.82-12.37)	0.001^∗∗^
	Latrine	235 (59.8)	100 (42.5)	135 (47.5)	1	
Source of drinking water	River	149 (38.9)	125 (83.9)	24 (16.1)	3.124 (1.325-7.36)	0.009^∗^
	Well	27 (7)	22 (81.5)	5 (18.5)	2.054 (0.47-8.95)	0.338
	Tap	207 (54)	92 (44.4)	115 (55.6)	1	
Shoe-wearing habit	Always	272 (66)	143 (52.6)	129 (47.4)	1	
	Sometimes	95 (24.8)	84 (88.4)	11 (11.6)	6.889 (3.51-13.48)	0.000^∗∗^
	Not at all	16 (4.2)	12 (75)	4 (25)	2.706 (0.851-8.60)	.092
Habits of washing fruits and vegetables before eating	Always	150 (21.4)	74 (49.3)	76 (50.7)	1	
	Sometimes	215 (56.1)	152 (70.7)	63 (29.3)	1.993 (0.32-12.22)	0.456
	Not at all	18 (4.7)	15 (83.3)	3 (16.7)	1.77 (0.77-4.04)	0.174
Hand washing habit after toilet use	Always	163 (42.6)	73 (44.8)	90 (55.2)	1	
	Sometimes	207 (54)	154 (74.4)	53 (25.6)	2.358 (1.047-5.31)	0.038^∗^
	Not at all	13 (3.4)	12 (92.3)	1 (7.7)	3.137 (0.19-49.61)	0.417
Raw meat-eating habit	Frequent	49 (13)	37 (75.5)	12 (24.5)	1.127 (0.31-4.079)	0.856
	Sometimes	177 (28.7)	110 (62.1)	67 (37.9)	0.470 (0.124-1.78)	0.267
	Not at all	157 (41)	94 (59.9)	63 (40.1)	1	
Ways of waste disposal	Burning	141 (23.8)	33 (23.4)	108 (76.6)	1	
	Burying	91 (23.8)	78 (85.7)	13 (14.3)	12.12 (4.24-34.62)	0.000^∗∗^
	Open dump	151 (39.4)	128 (84.8)	23 (15.2)	8.377 (3.55-19.75)	0.000^∗∗^
Fingernail cleanliness^@^	Clean	256 (66.8)	128 (50)	128 (50)	1	
	Not clean	127 (33.2)	111 (87.4)	16 (12.6)	4.2 (1.67-10.55)	0.002^∗^

Note: 1 = reference value; ^∗∗^statistically significant at *p* ≤ 0.001; ^∗^statistically significant at *p* < 0.05; AOR = adjusted odds ratio; multivariate regression model for grade level, family size, residence, family monthly income, father education, mother education, father occupation, mother occupation, place of defecation, source of drinking water, shoe-wearing habit, habit of eating unwashed fruits and vegetables, handwashing habit after toilet use, raw meat-eating habits, ways of waste disposal, and fingernails cleanliness for intestinal parasitic infections; @ = finger nail cleanliness was evaluated by observing whether nails are trimmed or not.

**Table 4:** Risk factors associated with *Ascaris lumbricoides* and hookworm infections.

Risk factors	*Ascaris lumbricoides* infection				
	Positive no. (%)	Negative no. (%)	Total no. (%)	AOR (95% CI)	*p* value
Age groups					
Childhood	4 (50)	4 (50)	8 (2.1)	2.621 (0.631-19.039)	0.341
Midchildhood	65 (20.3)	255 (79.7)	320 (83.5)	0.686 (0.317-1.405)	0.287
Early adolescent	18 (32.7)	37 (67.3)	55 (14.4)	1	
Residence					
Rural	46 (25.3)	136 (74.7)	182 (47.5)	0.96 (0.545-1.715)	0.908
Urban	41 (20.4)	160 (79.6)	201 (52.5)	1	
Family size					
2-4	12 (26.7)	33 (73.3)	45 (11.7)	2.706 (1.043-7.017)	0.041^∗^
5-7	51 (26.7)	140 (73.3)	191 (49.9)	1.695 (0.915-3.142)	
8-10	24 (16.3)	123 (83.7)	147 (38.4)	1	
Family monthly income (ETB)					
<800	57 (35)	106 (65)	163 (42.6)	2.004 (0.709-5.667)	0.190
800-2,000	23 (13.5)	147 (86.5)	170 (44.4)	1.035 (0.356-3.011)	0.950
>2,000	7 (14)	43 (86)	50 (13.1)	1	
Father education					
Primary school	60 (27.1)	161 (72.9)	221 (57.7)	1490 (0.000)	0.999
Secondary school and above	27 (16.7)	135 (83.3)	162 (40.5)	1486 (0.000)	0.999
College and above	-	7 (100)	7 (1.8)	1	
Father occupation					
Daily laborer	9 (60)	6 (40)	15 (3.9)	1.728 (0.284-10.512)	0.553
Farmer	73 (22)	259 (78)	332 (86.4)	0.719 (0.192-2.691)	0.624
Govt. employee	1 (6.2)	15 (93.8)	16 (4.2)	0.288 (0.020-4.065)	0.357
Merchant	4 (20)	16 (80)	20 (5.2)	1	
Place of defecation					
Toilet	37 (15.1)	198 (84.3)	235 (61.4)	1	
Open field	50 (33.8)	98 (66.2)	148 (38.6)	0.694 (0.505-1.841)	0.911
Drinking water sources					
River	49 (32.9)	100 (67.1)	149 (38.9)	1.675 (0.915-3.142)	0.112
Well	9 (33.3)	18 (66.7)	27 (7)	1.937 (0.704-5.332)	0.201
Pipe	29 (14)	178 (86)	207 (54)	1	
Fruit and vegetation washing habit before eating					
Not at all	7 (38.9)	11 (61.1)	18 (4.7)	1.422 (0.410-4.929)	0.579
Sometimes	58 (27)	157 (73)	215 (56.1)	1.141 (0.598-2.176)	0.689
Always	22 (14.7)	128 (85.3)	150 (39.2)	1	
Hand washing habit after toilet use					
Not at all	4 (30.9)	9 (69.2)	13 (3.4)	1.278 (0.283-5.764)	0.749
Sometimes	60 (29)	147 (71)	207 (54)	1.428 (0.756-2.698)	0.273
Always	23 (14.1)	140 (85.9)	163 (42.6)	1	
Ways of waste disposal					
Open damp	58 (38.4)	93 (61.6)	151 (39.4)	8.033 (3.022-21.355)	0.001^∗∗^
Burying	23 (25.3)	68 (74.7)	91 (23.8)	4.898 (1.722-13.929)	0.003^∗^
Burning	6 (4.3)	135 (95.7)		1	
Fingernail cleanliness					
Not clean	40 (31.5)	87 (68.5)	127 (33.2)	1.301 (0.727-2.328)	0.376
Clean	47 (18.4)	209 (81.6)	256 (66.8)	1	

Hookworm infection					
Family size					
2-4	5 (11.1)	40 (88.9)	45 (11.7)	0.513 (0.169-1.555)	0.238
5-7	43 (22.6)	148 (77.5)	191 (49.9)	1.112 (0.619-1.998)	0.722
8-10	31 (21.1)	116 (78.9)	147 (38.4)	1	
Family monthly income (ETB)					
<800	41 (25.2)	122 (74.8)	163 (42.6)	1.038 (0.375-2.873)	0.943
800-2,000	31 (18.2)	139 (81.8)	170 (44.4)	1.314 (0.484-3.562)	0.592
>2,000	7 (14)	43 (86)	50 (13.1)	1	
Mother education					
Primary school	76 (21.9)	271 (78.1)	347 (90.6)	4893 (0.000-000)	0.999
Secondary school	3 (9.4)	29 (90.6)	32 (8.4)	2958 (0.000-000)	0.999
College and above	-	4 (100)	4 (100)	1	
Father education					
Primary school	54 (24.4)	167 (75.6)	221 (57.7)	0.208 (0.021-2.027)	0.176
Secondary school	23 (14.8)	132 (85.2)	155 (40.4)	0.167 (0.017-1.646)	0.125
College and above	2 (28.6)	5 (71.4)	7 (1.8)	1	
Mother occupation					
Daily laborer	1 (33.3)	2 (66.7)	3 (0.8)	2.015 (0.103-39.620)	0.645
Housewife	76 (21.7)	274 (78.3)	350 (9.4)	1.685 (0.331-8.576)	0.530
Govt. employee	-	9 (100)	9 (2.3)	0.000 (0.000-0.000)	0.999
Merchant	2 (9.5)	19 (90.5)	21 (5.57)	1	
Place of defecation					
Open field	48 (32.4)	100 (67.6)	148 (38.6)	1.567 (0.812-3.022)	0.180
Toilet	31 (13.2)	204 (86.8)	235 (61.4)	1	
Source of drinking water					
River	40 (26.8)	109 (73.2)	149 (38.9)	1.329 (0.702-2.513)	0.382
Well	10 (37)	17 (63)	27 (7)	1.962 (0.716-5.380)	0.190
Pipe	29 (14)	178 (86)	207 (54)	1	
Shoe-wearing habit					
Not at all	9 (56.2)	7 (43.8)	16 (4.2)	8.346 (2.908-18.761)	0.001^∗∗^
Sometimes	35 (36.8)	60 (63.2)	95 (24.8)	3.648 (2.094-6.358)	0.001^∗∗^
Frequent	35 (12.9)	237 (87.1)	272 (71)	1	
Hand washing habit after toilet use					
Not at all	5 (38.5)	8 (61.5)	13 (3.4)	1.547 (0.408-5.867)	0.521
Sometimes	49 (23.7)	158 (76.3)	207 (54)	0.919 (0.495-1.706)	0.789
Always	25 (15.3)	138 (84.7)	163 (42.6)	1	
Ways of waste disposal					
Open damp	36 (23.8)	115 (76.2)	151 (39.4)	1.686 (0.761-3.736)	0.198
Burying	30 (33)	61 (67)	91 (23.8)	2.970 (1.290-6.838)	0.011^∗^
Burning	13 (9.2)	128 (90.8)	141 (36.8)	1	
Fingernail cleanliness					
Not clean	35 (27.6)	92 (72.4)	127 (33.2)	1.047 (0.585-1.872)	0.878
Clean	44 (17.2)	212 (82.8)	256 (66.8)	1	

Note: 1 = reference value; ^∗∗^statistically significant at *p* ≤ 0.001;^∗^statistically significant at *p* < 0.05, AOR = adjusted odds ratio; multivariate regression model for age, residence, family size, family monthly income, father education, father occupation, place of defecation, source of drinking water, habit of eating unwashed fruits and vegetables, handwashing habit after toilet use, ways of waste disposal, and fingernails cleanliness for *Ascaris lumbricoides* infection, and residence, family size, family monthly income, father education, mother education, mother occupation, place of defecation, source of drinking water, handwashing habit after toilet use, ways of waste disposal, and fingernails cleanliness for hookworm infection.

## Data Availability

The [some statistical analyses and STROBE Statement] data used to support the findings of this study are included within the supplementary information file(s).

## Supplementary Materials

Supplementary 1 Supplementary material file 1: the association of IPIs with potential risk factors (sociodemographic, behavioral, personal hygienic practices, and environmental sanitation) among students in the first-cycle primary school in Sasiga District, southwest Ethiopia, 2018/2019.

Supplementary 2 Supplementary material file 2: the association of the risk factors related to personal, behavioral, and hygienic practices with the detected intestinal parasites among students in the first-cycle primary school in Sasiga District, southwest Ethiopia, 2018/2019.

Supplementary 3 Supplementary material file 3: multivariate logistic regression analysis of potential risk factors associated with *Entameoba histolytica*/*dipar*, *Trichuris trichiura*, *Giardia intestinalis*, *Hymenolepis nana*, and *Schistosoma mansoni* infections among school children in Sasiga District, southwest Ethiopia, 2019.

Supplementary 4 Supplementary material file 4: STROBE Statement—Checklist of items that are included in the reports of this cross-sectional studies on the prevalence of IPIs among students in the first-cycle primary school in Sasiga District, southwest Ethiopia, 2018/2019. 22 STROBE checklists for cross-sectional studies were included here.
