# Supplementary material for: Prevalence of Intestinal Parasitic Infections and Associated Risk Factors among the First-Cycle Primary Schoolchildren in Sasiga District, Southwest Ethiopia
Source: J Parasitol Res. 2020 Mar 13;2020:8681247. doi: 10.1155/2020/8681247 (PMC7093910; doi:10.1155/2020/8681247)
Supplement: Supplementary 1 — Supplementary material file 1: the association of IPIs with potential risk factors (sociodemographic, behavioral, personal hygienic practices, and environmental sanitation) among students in the first-cycle primary school in Sasiga District, southwest Ethiopia, 2018/2019. [file 8681247.f1.docx]

**Supplementary file 1:** The association of IPIs with potential risk factors (socio-demographic, behavioral, personal hygienic practices and environmental sanitation) among students in Sasiga first cycle Primary School, Sasiga District, south-west Ethiopia, 2018/19

| Potential risk factors | | Number and percentage of parasite infected student | | |  |  |
| --- | --- | --- | --- | --- | --- | --- |
| Socio-demographic variables | |  |  |  |  |  |
| Variables | Categories | Total No.(%) | Positive No. (%) | Negative No.(%) | *X*^2^ | P-value |
| Grade level | 1 | 120(31.3) | 80(66.6) | 40(33.33) | 4.25 | 0.236 |
|  | 2 | 112(29.2) | 71(63.4) | 41(36.6) |  |  |
|  | 3 | 83(21.7) | 44(53) | 39(47) |  |  |
|  | 4 | 68(17.8) | 44(64.7) | 24(35.3) |  |  |
| Age groups | Childhood | 8(2.1) | 6(75) | 2(25) | 0.856 | 0.652 |
|  | Mid childhood | 320(83.5) | 197(61.6) | 123(38.4) |  |  |
|  | Early adolescent | 55(14.4 ) | 36(65.5) | 19(34.5) |  |  |
| Sex | Male | 187(48.8) | 117(62.6) | 70(37.4) | 0.004 | 0.948 |
|  | Female | 196(51.2) | 122(62.2) | 74(37.8) |  |  |
| Residence | Urban | 201(52.5) | 100(49.8) | 101(50.2) | 28.85 | 0.001^**^ |
|  | Rural | 182(47.5) | 139(76.4) | 43(23.6) |  |  |
| Family size | <4 | 45(11.75) | 23(51.1) | 22(48.9) | 5.243 | 0.073 |
|  | 5-7 | 191(49.9) | 129(67.5) | 62(32.5) |  |  |
|  | 8-10 | 147(38.38) | 87(59.2) | 60(40.8) |  |  |
| Family monthly  income(ETB) | <800 | 163(42.6) | 141(86.5) | 22(13.5) | 70.22 | 0.001^**^ |
|  | 800-2000 | 170(44.9) | 76(44.7) | 94(55.3) |  |  |
|  | >2000 | 50(13.1) | 22(44) | 28(56) |  |  |
| Father education | Primary school | 221(57.7) | 157(71.1) | 64(28.9) | 16.80 | 0.009^*^ |
|  | Secondary school & above | 162(42.3) | 82(50.6) | 80(49.4) |  |  |
| Mother education | Primary school | 347(90.6) | 224(64.5) | 123(35.5) | 7.2 | 0.007^*^ |
|  | Secondary school & above | 36(9.44) | 15(41.7) | 21(58.3) |  |  |
| Father occupation | Daily laborer | 15(3.9) | 14(93.3) | 1(6.7) | 12.79 | 0.005* |
|  | Farmer | 332(86.7) | 208(62.6) | 124(37.4) |  |  |
|  | Merchant | 20(5.2) | 12(60) | 8(40) |  |  |
|  | Govt. employee | 16(4.2) | 5(31.3) | 11(69.7) |  |  |
| Mother occupation | House wife | 350(91.4) | 229(65.4) | 121(34.6) | 25.67 | 0.001** |
|  | Govt. employee | 9(1.3) | 0 | 9 |  |  |
|  | Merchant | 21(5.5 ) | 7 (33.3) | 14(66.7) |  |  |
|  | Daily laborer | 3(1.6) | 3 | -- |  |  |
| Behavioral factors, hygienic practice and environmental variables | |  |  |  |  |  |
| Variables | Categories | Total(%) | Positive(%) | Negative(%) | *X*^2^ | P-value |
| Place of defecation | Latrine | 235(59.8) | 100(42.5) | 135(47.5) | 102.121 | 0.0001** |
|  | Open field | 148(38.6) | 139(93.9) | 9(6.1) |  |  |
| Source of drinking water | Tap | 149(38.9) | 92(44.4) | 115(55.6) | 61.971 | 0.001** |
|  | Well | 27(7) | 22(81.5) | 5(18.5) |  |  |
|  | River | 207(54) | 125(83.9) | 24(16.1) |  |  |
| Shoe-wearing habit | Always | 272(66) | 143(52.6) | 129(47.4) | 39.693 | 0.002* |
|  | Sometimes | 95(24.8) | 84(88.4) | 11(11.6) |  |  |
|  | Not at all | 16(4.2) | 12(75) | 4(25) |  |  |
| Hand washing habits before eating fruit &vegetables | Always | 150(21.4) | 74(49.3) | 76(50.7) | 22.929 | 0.001** |
|  | Sometimes | 215(56.1) | 152(70.7) | 63(29.3) |  |  |
|  | Not at all | 18(4.7) | 15(83.3) | 3(16.7) |  |  |
| Handwashing habit after toilet use | Always | 163(42.6) | 73(44.8) | 90(55.2) | 39.209 | 0.002* |
|  | Sometimes | 207(54) | 154(74.4) | 53(25.6) |  |  |
|  | Not at all | 13(3.4) | 12(92.3) | 1(7.7) |  |  |
| Raw meat-eating habit | Frequent | 49(13) | 37(75.5) | 13(24.5) | 3.65 | 0.161 |
|  | Sometimes | 177(28.7) | 110(62.1) | 67(37.9) |  |  |
|  | Not at all | 157(41) | 94(59.9) | 63(40.1) |  |  |
| Ways of waste disposal | Burning | 141(23.8) | 33(23.4) | 108(76.6) |  |  |
|  | Burying | 91 (23.8) | 78(85.7) | 13(14.3) | 144.672 | 0.001** |
|  | Open dump | 151(39.4) | 128(84.8) | 23(15.2) |  |  |
| Fingernail cleanliness | Clean | 256(66.8) | 128(50) | 128(50) | 50.613 | 0.001** |
|  | Not clean | 127(33.2) | 111(87.4) | 16(12.6) |  |  |

**=statistically significant at P≤0.001*=statistically significant at p<0.05
