# Supplementary material for: Prevalence of Intestinal Parasitic Infections and Associated Risk Factors among the First-Cycle Primary Schoolchildren in Sasiga District, Southwest Ethiopia
Source: J Parasitol Res. 2020 Mar 13;2020:8681247. doi: 10.1155/2020/8681247 (PMC7093910; doi:10.1155/2020/8681247)
Supplement: Supplementary 2 — Supplementary material file 2: the association of the risk factors related to personal, behavioral, and hygienic practices with the detected intestinal parasites among students in the first-cycle primary school in Sasiga District, southwest Ethiopia, 2018/2019. [file 8681247.f2.docx]

**Supplementary** file 2: Association of personal, behavioral, and hygienic practice risk factors with intestinal parasites among students in Sasiga first cycle Primary School, Sasiga District, south-west Ethiopia, 2018/19

| Parasites | Risk factors |  |  |  |  |  |
| --- | --- | --- | --- | --- | --- | --- |
|  | Place of defecation No.(%) | |  |  |  |  |
|  | Open field | Toilet | - | Total.No(%) | X^2^ | p-value |
| *A.lumbricoides* | 50(33.8) | 37(15.7) | - | 87(22.7) | 16.832 | 0.001** |
| Hookworms | 48(32.4) | 31(13.2) | - | 79(20.6) | 20.534 | 0.001** |
| *E.histolytica/dispar* | 24(16.2) | 7(3) | - | 31(8.1) | 31.391 | 0.001** |
| *G.intestinalis* | 16(10.8) | 9(3.8) | *-* | *25(6.5)* | 7.253 | 0.007 |
| *H.ana* | 11(7.4) | 11(4.7) | - | 22(5.7) | 1.270 | 0.26 |
| *S.mansoni* | 11(7.4) | 6(2.6) | - | 17(4.4) | 5.097 | 0.024* |
| *T.trichuira* | 17(11.5) | 12(5.1) | - | 29(7.6) | 5.282 | 0.022* |
|  | Source of drinking water No.(%) | | |  |  |  |
|  | River | Well | Pipe | Total | X^2^ | p-value |
| *A.lumbricoides* | 49(32.9) | 9(33.3) | 29(14) | 87(22.7) | 19.450 | 0.001** |
| Hookworms | 40(26.8) | 10(37) | 29(14) | 79(20.6) | 13.497 | 0.001** |
| *E.histolytica/dispar* | 23(15.4) | 1(3.7) | 7(3.4) | 31(8.1) | 17.677 | 0.001** |
| *G.intestinalis* | 10(6.7) | 3(11.1) | 12(5.8) | 25(6.5) | 1.119 | 0.572 |
| *H.ana* | 11(7.4) | 1(3.7) | 10(4.8) | 22(5.7) | 1.265 | 0.531 |
| *S.mansoni* | 12(8.1) | 1(3.7) | 4(1.9) | 17(4.4) | 7.691 | 0.021* |
| *T.trichuira* | 13(3.4) | 1(0.3) | 15(3.9) | 29(7.6) | 0.892 | 0.640 |
|  | Shoes wearing habit No.(%) | | |  |  |  |
|  | Not at all | Sometimes | Frequent | Total | X^2^ | p-value |
| *A.lumbricoides* | 3(18.8) | 24(25.3) | 60(22.1) | 87(22.7) | 0.560 | 0.755 |
| Hookworms | 9(56.2) | 35(36.8) | 35(12.9) | 79(20.6) | 37.661 | 0.001** |
| *E.histolytica/dispar* | - | 14(14.7) | 17(6.2) | 31(8.1) | 8.288 | 0.016* |
| *G.intestinalis* | 2(12.5) | 6(6.3) | 17(6.2) | 25(6.5) | 0.977 | 0.614 |
| *H.ana* | - | 9(9.5) | 13(4.8) | 22(5.7) | 3.883 | 0.143 |
| *S.mansoni* | - | 6(6.3) | 11(4) | 17(4.4) | 7.691 | 0.021* |
| *T.trichuira* | - | 11(11.6) | 18(6.6) | 29(7.6) | 3.844 | 0.146 |
|  | Fruit and vegetable washing habit before eating No.(%) | | | |  |  |
|  | Not at all | Sometimes | Always | Total | X^2^ | p-value |
| *A.lumbricoides* | 7(38.9) | 58(27) | 22(14.7) | 87(22.7) | 10.447 | 0.005* |
| Hookworms | 4(22.2) | 48(22.3) | 27(18) | 79(20.6) | 1.039 | 0.595 |
| *E.histolytica/dispar* | 3(18.7) | 18(8.4) | 10(6.7) | 31(8.1) | 2.211 | 0.331 |
| *G.intestinalis* | 1(5.6) | 14(6.5) | 10(6.7) | 25(6.5) | 0.033 | 0.984 |
| *H.ana* | 1(5.6) | 16(7.4) | 5(3.3) | 22(5.7) | 2.756 | 0.252 |
| *S.mansoni* | - | 10(4.7) | 7(4.7) | 17(4.4) | 0.877 | 0.645 |
| *T.trichuira* | 4(22) | 17(7.9) | 8(5.3) | 29(7.6) | 6.629 | 0.036* |
|  | Hand washing habit after toilet use No.(%) | | |  |  |  |
|  | Not at all | Sometimes | Always | Total | X^2^ | p-value |
| *A.lumbricoides* | 4(1) | 60(15.7) | 23(6) | 87(22.7) | 11.991 | 0.002* |
| Hookworms | 5(1.3) | 49(12.8) | 25(6.5) | 79(20.6) | 6.483 | 0.039* |
| *E.histolytica/dispar* | 1(0.3) | 21(5.5) | 9(2.3) | 31(8.1) | 2.235 | 0.327 |
| *G.intestinalis* | 1(0.3) | 14(3.7) | 10(2.6) | 25(6.5) | 0.089 | 0.957 |
| *H.ana* | 1(0.3) | 15(2.9) | 6(1.6) | 22(5.7) | 2.235 | 0.327 |
| *S.mansoni* | - | 12(3.1) | 5(0.5) | 17(4.4) | 2.227 | 0.328 |
| *T.trichuira* | 2(0.5) | 21(5.5) | 6(1.6) | 29(7.6) | 6.618 | 0.037* |
|  | Raw meat eating habit No.(%) | | |  |  |  |
|  | Not at all | Sometimes | Frequent | Total | X^2^ | p-value |
| *A.lumbricoides* | 38(24.2) | 39( 22) | 10(20.4) | 87(22.7) | 0.394 | 0.821 |
| Hookworms | 29(18.5) | 42(23.7) | 8(16.3) | 79(20.6) | 2.039 | 0.361 |
| *E.histolytica/dispar* | 14(8.9) | 13(7.3) | 4(8.2) | 31(8.1) | 0.277 | 0.871 |
| *G.intestinalis* | 11(7) | 11(6.2) | 3(6.1) | 25(6.5) | 0.101 | 0.951 |
| *H.ana* | 4(2.5) | 6(3.4) | 12(24.5) | 22(5.7) | 36.577 | 0.001** |
| *S.mansoni* | 6(3.8) | 8(4.5) | 3(6.1) | 17(4.4) | 0.471 | 0.790 |
| *T.trichuira* | 12(7.6) | 9(5.1) | 8(16.3) | 29(7.6) | 6.932 | 0.031 |
|  | Ways of waste disposal No.(%) | | |  |  |  |
|  | Open damp | Burying | Burning | Total | X^2^ | p-value |
| *A.lumbricoides* | 58(38.8) | 23(25.3) | 6(4.3) | 87(22.7) | 48.898 | 0.001** |
| Hookworms | 36(23.8) | 30(33) | 13(13) | 79(20.6) | 20.623 | 0.001** |
| *E.histolytica/dispar* | 18(11.9) | 7(7.7) | 6(4.3) | 31(8.1) | 5.785 | 0.055 |
| *G.intestinalis* | 10(6.6) | 11(12.1) | 4(2.8) | 25(6.5) | 7.761 | 0.021* |
| *H.ana* | 13(8.6) | 3(3.3) | 6(4.3) | 22(5.7) | 3.873 | 0.144 |
| *S.mansoni* | 9(6) | 5(5.5) | 3(2.1) | 17(4.4) | 2.839 | 0.242 |
| *T.trichuira* | 17(11.3) | 10(11) | 2(14) | 29(7.6) | 12.079 | 0.002* |
|  | Finger and nail cleanness No.(%) | | |  |  |  |
|  | Not clean (%) | clean (%) | - | Total | X^2^ | p-value |
| *A.lumbricoides* | 40(31.5) | 47(18.4) | - | 87(22.7) | 8.345 | 0.004* |
| Hookworms | 35(27.6) | 44(17.2) | - | 79(20.6) | 5.577 | 0.018* |
| *E.histolytica/dispar* | 18(14.2) | 13(5.1) | - | 31(8.1) | 9.440 | 0.002* |
| *G.intestinalis* | 13(10.2) | 12(4.7) |  | 25(6.5) | 4.284 | 0.038* |
| *H.nana* | 10(7.9) | 12(4.7) | - | 22(5.7) | 1.592 | 0.207 |
| *S.mansoni* | 9(7.1) | 8(3.1) | - | 17(4.4) | 3.141 | 0.076 |
| *T.trichuira* | 18(14.2) | 11(4.3) | - | 29(7.6) | 11.831 | 0.001** |

**=statistically significant at P≤0.001*=statistically significant at p<0.05
