# Supplementary material for: Prevalence of Intestinal Parasitic Infections and Associated Risk Factors among the First-Cycle Primary Schoolchildren in Sasiga District, Southwest Ethiopia
Source: J Parasitol Res. 2020 Mar 13;2020:8681247. doi: 10.1155/2020/8681247 (PMC7093910; doi:10.1155/2020/8681247)
Supplement: Supplementary 3 — Supplementary material file 3: multivariate logistic regression analysis of potential risk factors associated with Entameoba histolytica/dipar, Trichuris trichiura, Giardia intestinalis, Hymenolepis nana, and Schistosoma mansoni infections among school children in Sasiga District, southwest Ethiopia, 2019. [file 8681247.f3.docx]

Supplementary file 3. Multivariate logistic regression analysis of potential risk factors associated with *Entameoba histolytica*, *Trichuris trichiura, Giardia intestinalis, Hymenolepsis nana*, and *Schistosoma mansoni* infections among school children in Sasiga District, south-west Ethiopia, 2019

| Risk factors | *Entameoba histolytica* infection | | | | |
| --- | --- | --- | --- | --- | --- |
|  | Positive No.(%) | Negative No. (%) | Total No.(%) | AOR (95% CI.) | P-value |
| Age |  |  |  |  |  |
| Childhood | 1(12.5) | 7(87.5) | 8(2.1) | 15.781(0.536,464.461) | 0.101 |
| Mid childhood | 29(9.1) | 291(90.9) | 320(83.5) | 8.503(0.963,75.078 | 0.054 |
| Early adolescent | 1(1.8) | 54(98.2) | 55(14.1) | 1 |  |
| Residence |  |  |  |  |  |
| Rural | 11(6) | 171(94) | 182(47.5) | 0.507(0.194,1.321) | 0.164 |
| Urban | 20(10) | 181(90) | 201(52.5) | 1 |  |
| Family monthly income(ETB) |  |  |  |  |  |
| ≤800 | 21(12.9) | 142(87.1) | 163(34.5) | 1.407 (0. 258,7.679) | 0.693 |
| 800-2000 | 8(4.7) | 162(95.3) | 170(44.4) | 0.766(0.134,4.367) | 0.764 |
| ≥2000 | 2(4) | 48(96) | 50(13) | 1 |  |
| Father educational status |  |  |  |  |  |
| Primary school | 25(11.3) | 196(88.7) | 221(57.7) | 0.705(0.000, 0.000) | 1.00 |
| Secondary school | 6(3.9) | 149(96.1) | 155(40.7) | 0.350(0.000, 0.000) | 1.00 |
| College and above | - | 7(100) | 7(1.8) | 1 |  |
| Father occupation |  |  |  |  |  |
| Daily laborer | 3(20) | 12(80) | 15(3.9) | 0.426(0.014,13.266) | 0.67 |
| Farmer | 27(8.1) | 305(91.9) | 332(86.7) | 1.121(0.083,15.130) | 0.931 |
| Govt. employee | - | 16(100) | 16(4.2) | 0.000(0.000,0.000) | 0.999 |
| Merchant | 1(5) | 9(95) | 20(5.2) | 1 |  |
| Mother occupation |  |  |  |  |  |
| Daily laborer | 2(66.7) | 1(33.3) | 3(0.8) | 17.644(0.065, 482.752) | 0.316 |
| House wife | 27(7.7) | 323(92.3) | 350(91.4) | 0.258(0.033,2.022) | 0.197 |
| Govt. employee | - | 9(100) | 9(2.3) | 0.000(0.000, 0.000) | 0.999 |
| Merchant | 2(9.5) | 19(90.5) | 21(5.5) | 1 |  |
| Place of defecation |  |  |  |  |  |
| Open field | 24(16.2) | 124(83.8) | 148(38.6) | 4.470(1.261,15.842) | 0.020* |
| Toilet | 7(3) | 228(97) | 235(61.4) | 1 |  |
| Water source |  |  |  |  |  |
| River | 23(15.4) | 126(84.6) | 149(38.9) | 2.932(1.024) | 0.045* |
| Well water | 1(3.7) | 26(96.3) | 27(7) | 0.322(0.020,3.780) | 0.367 |
| Pipe | 7(3.4) | 200(9.6) | 207(54) | 1 |  |
| Shoes-wearing habit |  |  |  |  |  |
| Not at all | - | 16(85.3) | 16(4.2) | 0.998(0.000.0.000) | 0.998 |
| Sometimes | 14(14.7) | 81(8.1) | 32(8.4) | 0.870(0.924,0.350) | 0.870 |
| Frequent | 17(6.2) | 255(93.8) | 272(71) | 1 |  |
| Ways of waste disposal |  |  |  |  |  |
| Open damp | 18(11.8) | 133(88.1) | 151(39.4) | 0.873(0.239,3.188) | 0.837 |
| Burying | 7(7.7) | 84(92.3) | 91(10.7) | 0.635(0.144,2.791) | 0.548 |
| Burning | 6(4.3) | 135(95.7) | 141(36.8) | 1 |  |
| Fingernail cleanliness |  |  |  |  |  |
| Not clean | 18(14.2) | 109(85.8) | 127(33.2) | 2.355(0.920,6.027) | 0.548 |
| Clean | 13(5.1) | 243(94.9) | 256(66.1) | 1 |  |
| Family size |  |  |  |  |  |
| Risk factors | *Trichuris trichiura* infection | | |  |  |
|  | Positive No.(%) | Negative No. (%) | Total No. (%) | AOR (95% CI.) | P-value |
| Residence |  |  |  |  |  |
| Rural | 17(11.5) | 13(88.5) | 182(47.5) | 4.968(1.672,14.762) | 0.004* |
| Urban | 12(5.1) | 223(94.9) | 201(52.1) | 1 |  |
| Family monthly income(ETB |  |  |  |  |  |
| ≤800 | 19(11.7) | 144(88.3) | 163(42.6) | 3.580(0.376,34.762) | 0.267 |
| 800-2000 | 9(5.3) | 161(94.7) | 170(44.4) | 3.477(0.368,32.828) | 0.277 |
| ≥2000 | 1(2) | 49(98) | 50(13.1) | 1 |  |
| Father occupation |  |  |  |  |  |
| Daily laborer | 5(33.3) | 10(66.7) | 15(3.9) | 4286 (0.000,0.000) | 0.195 |
| Farmer | 23(6.2) | 309(93.1) | 332(86.7) | 4290 (0.000,0.000) | 0.999 |
| Govt. employee | 1(6.2) | 15(93.8) | 16(4.2) | 7986(0.000.0.000) | 0.998 |
| Merchant | - | 20(100) | 20(5.2) | 1 |  |
| Place of defecation |  |  |  |  |  |
| Open Field | 17(58.6) | 131(88.5) | 148(38.6) | 1.265(0.485,3.303) | 0.631 |
| Toilet | 12(41.4) | 223(94.9) | 235(61.4) | 1 |  |
| Shoes-wearing habit |  |  |  |  |  |
| Not at all | - | 16(100) | 16(4.2) | 0.000(0.000,0.000) | 0.998 |
| Sometimes | 11(11.6) | 84(88.4) | 95(24.8) | 0.994(0.384,2.577) | 0.990 |
| frequent | 18(6.6) | 254(93.4 ) | 272(71) | 1 |  |
| Unwashed Fruit and vegetables eating habit |  |  |  |  |  |
| Handwashing habit after toilet use |  |  |  |  |  |
| Not at all | 2(15.4) | 11(84.6) | 13(3.4) | 2.361(0.313,17.806) | 0.405 |
| Sometimes | 21(10.1) | 186(89.9) | 207(54) | 1.898(0.646,5.580) | 0.244 |
| Always | 6(3.7) | 157(96.3) | 163(42.6) | 1 |  |
| Raw meat-eating habit |  |  |  |  |  |
| Not at all | 12(7.6) | 145(92.4) | 157(41) | 0.760(0.234,2.469) | 0.648 |
| Sometimes | 9(5.1) | 168(94.9) | 177(46.2) | 0.254(0.075,0.863) | 0.028* |
| Frequent | 8(13.3) | 41(83.7) | 49(12.6) | 1 |  |
| Ways of waste disposal |  |  |  |  |  |
| Open damp | 17( 11.3) | 134(88.7) | 151(39.4) | 3.912(0.762,20.090) | 0.102 |
| Burying | 10( 11 ) | 81(89) | 91(23.8) | 4.792(0.891,25.785) | 0.068 |
| Burning | 2(1.4) | 139(98.6) | 141(36.8) | 1 |  |
| Fingernail cleanliness |  |  |  |  |  |
| Not clean | 18(14.2) | 109(85.8) | 127(33.2) | 2.378(0.962,5.785) | 0.061 |
| Clean | 11(4.3) | 245(95.7) | 256(66.8) | 1 |  |
| Risk factors | *Hymenolepsis nana* infection | |  |  |  |
| sex | Positive No.(%) | Negative No. (%) | Total No. (%) | AOR (95% CI.) | P-value |
| Female | 8(4.1) | 188(95.9) | 196(51) | 1 |  |
| Male | 14(7.5) | 173(92.5) | 187(49) | 2.44(0.747,7.458) | 0.118 |
| Residence |  |  |  |  |  |
| Rural | 22(12.1) | 201(87.9) | 182(47.5) | 1835(0.000,0.000) | 0.994 |
| Urban | - | 201(100) | 201)52.5) | 1 |  |
| Family monthly income(ETB) |  |  |  |  |  |
| <800 | 14(3.7) | 149(38.9) | 163(42.5) | 5.554(0.883,34.949) | 0.068 |
| 800-2000 | 6(1.6) | 164(42.8) | 170(44.4) | 2.981(0.00. 0.000) | 0.255 |
| >2000 | 2()0.2) | 48(12.5) | 50(13.1) | 1 |  |
| Mother occupation |  |  |  |  |  |
| Shoes-wearing habit |  |  |  |  |  |
| Not at all | - | 16(100) | 16(4.2) | 0.000(0.000,0.000) | 0.998 |
| Sometimes | 9(9.5) | 86(90.5) | 95(24.8) | 2.642(0.856,8.154) | 0.091 |
| Frequent | 13(4.8) | 259(95.2) | 272(71) | 1 |  |
| Raw meat-eating habit |  |  |  |  |  |
| Not at all | 4(2.5) | 153(97.5) | 157(41) | 0.118(0.030,0.460) | 0.002* |
| Sometimes | 6(3.4) | 171(96.6) | 177(46.2) | 0.114(0.034,0.386) | 0.000** |
| Frequent | 12(24.5) | 37(75.5) | 49(12.8) | 1 |  |
| Ways of waste disposal |  |  |  |  |  |
| Open damp | 13(8.6) | 138(91.4) | 151(39.4) | 0.799(0.220,2.906) | 0.733 |
| Burying | 3(3.3) | 88(96.7) | 91(23.8) | 0.236(0.040,1.401) | 0.112 |
| Burning | 6(4.3) | 135(95.7) | 141(36.8) | 1 |  |
| Fingernail cleanliness |  |  |  |  |  |
| Not clean | 10(7.9) | 117(92.1) | 127(33.2) | 1.071(0.352,3.261) | 0.904 |
| Clean | 12(4.7) | 244(95.3) | 256(66.8) | 1 |  |
| Risk factors | *Giardia intestinalis* infection |  |  |  |  |
| Grade level | Positive No.(%) | Negative No. (%) | Total No. (%) | AOR (95% CI.) | P.value. |
| 1 | 10(8.3) | 110(91.7) | 120(31.33) | 3.103(0.637,15.124) | 0.161 |
| 2 | 12(10.7) | 100(89.2) | 112(29.24) | 4.670(0.972,22.442) | 0.54 |
| 3 | 1 | 82(98.8) | 83(21.7) | 0.5.8(0.044,5.86) | 0.587 |
| 4 | 2 | 66(97.1) | 68(17.75) | 1 |  |
| Residence |  |  |  |  |  |
| Rural | 15(8.2) | 167(91.8) | 182(47.5) | 1.506(0.636,3.565) | 0.352 |
| Urban | 10(5) | 191(95) | 201(52.5) | 1 |  |
| Place of defecation |  |  |  |  |  |
| Open field | 16(10.8) | 132(89.2) | 148(38.6) | 2.222(0.865,5.704) | 0.097 |
| Latrine | 9(3.8) | 226(96.2) | 235(61.4) | 1 |  |
| Ways of Waste disposal |  |  |  |  |  |
| Open damp | 10(6.6) | 141(93.4) | 151(39.4) | 1.262(0.343,4.629) | 0.727 |
| Burying | 11(12.1) | 80(87.9) | 91(23.8) | 2.644(0.732,9.559) | 0.138 |
| Burning | 4(2.8) | 137(97.2) | 141(36.8) | 1 |  |
| Fingernail cleanliness |  |  |  |  |  |
| Not clean | 13(3.4) | 114(29.8) | 127(33.2) | 1.644(0.692,3.903) | 0.260 |
| Clean | 12(3.1) | 244(63.7) | 256(66.8) | 1 |  |
| Risk factors | *Schistosoma mansoni* infection |  |  |  |  |
| Age | Positive No.(%) | Negative No. (%) | Total No. (%) | AOR(95% CI.) | P-value. |
| 5 and less | - | 8(100) | 8(2.1) | 0.000(1.169,9.777) | 0.999 |
| 6-11 | 11(3.4) | 309(96.6) | 320(83.6) | 0.308(0.103,0.922) | 0.035* |
| 12-18 | 6(10.8) | 49(89.1) | 55(14.4) | 1 |  |
| Place of defecation |  |  |  |  |  |
| Open field | 11(7.4) | 137(92.6) | 148(38.6) | 1.735(0.504,5.922) | 0.383 |
| Toilet | 6(2.6) | 229(97.4) | 235(61.4) | 1 |  |
| Source of drinking water |  |  |  |  |  |
| River | 12(8.1) | 137(91.9) |  | 2.742(0.776,9.689) | 0.117 |
| Well | 1(3.7) | 26(96.3) | 149(38.9) | 1,355(0.133,13.767) | 0.797 |
| Pipe | 4(1.9) | 203(98.1) | 207(54) | 1 |  |
| Shoes-wearing habit |  |  |  |  |  |
| Not at all | - | 16(100) | 16(4.2) | 0.000(0.000,0.000) | 0.998 |
| Sometimes | 6(6.3) | 89(93.7) | 95(24.8) | 1.008(0.329,3.089) | 0.998 |
| Frequent | 11(4) | 261(96) | 272(71) | 1 |  |
| Ways of waste disposal |  |  |  |  |  |
| Open damp | 9(6) | 142(94) | 151(39.4) | 1.446(0.324,6.454) | 0.629 |
| Burying | 5(5.5) | 86(94.5) | 91(23.8) | 1.410(0.275,7.224) | 0.680 |
| Burning | 3(2.1) | 138(97.9) | (141(36.8) | 1 |  |
| Fingernail cleanliness |  |  |  |  |  |
| Not clean | 9(7.1) | 118(92.9) | 127(33.2) | 1.643(0.568,4.751) | 0.359 |
| Clean | 8(3.1) | 248(96.9) | 256(66.8) | 1 |  |

**=statistically significant at P≤0.001;*=statistically significant at p<0.05
